# Frequency of different types of facial melanoses referring to the Department of Dermatology and Venereology, Nepal Medical College and Teaching Hospital in 2019, and assessment of their effect on health-related quality of life

**DOI:** 10.1186/s12895-020-00100-3

**Published:** 2020-08-03

**Authors:** Bibush Amatya, Anil Kumar Jha, Shristi Shrestha

**Affiliations:** Department of Dermatology and Venereology, Nepal Medical College and Teaching Hospital (NMCTH), Attarkhel, Jorpati, Kathmandu, Nepal

**Keywords:** Facial melanoses, Facial pigmentary disorders, Quality of life, DLQI, DLQI comparison, Nepal, Melasma with steroid induced rosacea-like dermatitis

## Abstract

**Background:**

Abnormalities of facial pigmentation, or facial melanoses, are a common presenting complaint in Nepal and are the result of a diverse range of conditions.

**Objectives:**

The objective of this study was to determine the frequency, underlying cause and impact on quality of life of facial pigmentary disorders among patients visiting the Department of Dermatology and Venereology, Nepal Medical College and Teaching Hospital (NMCTH) over the course of one year.

**Methods:**

This was a cross-sectional study conducted at the Department of Dermatology and Venereology, NMCTH. We recruited patients with facial melanoses above 16 years of age who presented to the outpatient department. Clinical and demographic data were collected and all the enrolled participants completed the validated Nepali version of the Dermatology Life Quality Index (DLQI).

**Results:**

Between January 5, 2019 to January 4, 2020, a total of 485 patients were recruited in the study. The most common diagnoses were melasma (166 patients) and post acne hyperpigmentation (71 patients). Quality of life impairment was highest in patients having melasma with steroid induced rosacea-like dermatitis (DLQI = 13.54 ± 1.30), while it was lowest in participants with ephelides (2.45 ± 1.23).

**Conclusion:**

Facial melanoses are a common presenting complaint and lead to substantial impacts on quality of life. Accurate diagnosis and management can prevent or treat many facial melanoses, including those that lead to substantial loss of quality of life, such as melasma with steroid induced rosacea-like dermatitis. Health care systems in low and middle-income countries should dedicate resources to the identification, prevention and treatment of these conditions to improve quality of life.

## Background

Facial melanoses are a diverse range of disorders resulting in abnormal pigmentation of the facial region. Primary dermatologic disorders of pigmentation include, but are not limited to, melasma, ashy dermatosis, ephelides, solar lentigo, Riehl’s melanosis, peribuccal pigmentation of Brocq, naevus of Ota, Becker’s naevus, Hori’s naevus, ochronosis, frictional melanosis, actinic lichen planus, DLE and various types of postinflammatory hyperpigmentation [1, 2] Other conditions that may result in abnormal pigmentation of the facial region include Mongolian spots, late-stage failure of cardiopulmonary or renal system and drug/heavy metal induced pigmentation such as with iron, silver, gold, chloramphenicol, tetracycline, amiodarone, pirfenidone, antimalarials and antipsychotics [3]. Systemic disorders including Addison disease, haemochromatosis and porphyria cutanea tarda can also lead to abnormal pigmentation of face [4]. The diagnosis and differentiation of these conditions are based on history and clinical examination supplemented in some cases by Wood’s lamp and histopathological evaluation.

Although all these melanoses are associated with facial darkening, the heterogeneity of these conditions results in different levels of disease related impacts on quality of life. The dermatology life quality index (DLQI) is an assessment tool that can be used to measure the physical, psychological and social burden of dermatological disorders [5]. Although several other indices have been developed to measure disease specific impact on quality of life, the DLQI remains a simple and effective tool to measure and compare the impact of dermatological disorders.

A number of studies have investigated the prevalence of pigmentary disorders as part of an overall assessment of the burden of skin diseases in Nepal, where pigmentary disorders constitute 5–30% of the total burden of skin diseases [6–9]. Several studies have also focused on specific pigmentary disorders like melasma, vitiligo and their psychosocial and cosmetic challenges [10]. These studies suggest that pigmentary disorders are associated with significant social, psychological and cosmetic challenges because of their visibility on exposed areas of the body and because of the stigma associated with pigmentary alteration in Nepal and the Indian subcontinent [11, 12].

Very few published studies from Nepal have investigated the overall patterns of facial melanoses and assessed their impact on quality of life. Data on disease prevalence and its impact on quality of life provide a good measure of health status. We investigated the pattern of facial pigmentary disorders and their impact on quality of life among patients presented to a dermatology clinic at a tertiary care centre in Nepal in order to describe the burden and impact of these conditions. We believe the information obtained from this study can help in planning and allocating resources for the prevention and management of these conditions.

## Methods

This was a cross sectional study conducted at the Department of Dermatology and Venereology, Nepal Medical College and Teaching Hospital (NMCTH), Kathmandu, Nepal. The study subjects included all patients aged 16 years and over with a facial melanosis who visited the dermatology outpatient department between January 5, 2019 to January 4, 2020. All participants provide written informed consent for participation. The Institutional Review Committee (IRC) of NMCTH granted approval to conduct the study (reference number 029–075/076).

At enrolment, all participations filled a clinical proforma to obtain information on patient age, sex, diagnosis, duration and involved sites. The participants then completed a translated and validated Nepali version of the DLQI questionnaire to measure the impact of these facial melanoses on the quality of their lives. The translated and validated Nepali version of the DLQI has previously been used in other studies conducted in Nepal [10]. The DLQI questionnaire comprises 10 questions, each with four possible answers scored from zero to three, covering the last one week of the patient’s life. The sum of these scores is then calculated as the total DLQI score [5]. The scores range from 0 (no impact on quality of life) to 30 (highest level of impact). The interpretation of the DLQI score is as follows: 0–1 = No effect on patient’s life, 2–5 = Small effect, 6–10 = Moderate effect, 11–20 = Very large effect, 21–30 = Extremely large effect [5].

The investigators maintained strict confidentiality throughout the study. All participants who recorded a DLQI score greater than 10 were offered counselling by a trained counsellor of the Department of Psychology. The data were uploaded into a secure password protected database using Microsoft Excel.

### Statistical analysis

Data analysis was performed with the Statistical Package for Social Sciences (SPSS) version 16. Descriptive statistics were utilized to compute the mean and standard deviation of age and DLQI. Kolmogorov-Smirnov and Shapiro-Wilk tests were used to check for normality of DLQI. Mann-Whitney U test was used to compare the mean DLQI scores in participants with melasma and melasma with steroid induced rosacea-like dermatitis. Kruskal-Wallis H test was used to compare the mean DLQI scores in three or more conditions. Results were considered statistically significant at an alpha of 5% (*p* ≤ 0.05).

## Results

A total of 672 patients were eligible to participate in the study and 485 provided informed consent. Most participants were female (381, 78.56%). The mean age of the participants was 32.7 ± 12.3 years with a range of 67 years (16–83 years). The highest percentage of participants (71.3%) belonged to the age group 16–35 years.

The most common diagnosis was melasma (166 patients, 43%) followed by acne induced hyperpigmentation (71 patients, 18%). There were an additional 38 patients with melasma also having features of steroid induced rosacea-like dermatitis (Table 1). The conditions least commonly observed were Hori’s naevus (1 patient), verrucous epidermal naevus (1 patient), ochronosis secondary to use of hydroquinone (1 patient) and peribuccal pigmentation of Brocq (2 patients). Female predominance was observed in all facial melanoses except for Becker’s naevus, where all the participants were male (Table 1).

**Table 1:** Pattern and gender distribution of facial melanoses

Diagnosis	Number	Males	Females
Melasma	166 (34.23%)	27 (16.27%)	139 (83.73%)
Post-inflammatory hyperpigmentation secondary to acne	71 (14.64%)	25 (35.21%)	46 (64.79%)
Melasma with steroid induced rosacea-like dermatitis	38 (7.84%)	0	38 (100%)
Post-inflammatory hyperpigmentation due to other causes	33 (6.80%)	10 (30.3%)	23 (69.7%)
Ashy dermatosis	26 (5.36%)	3 (11.54%)	23 (88.46%)
DLE	26 (5.36%)	6 (23.08%)	20 (76.92%)
Compound naevus	25 (5.15%)	8 (32%)	17 (68%)
Ephelides	20 (4.12%)	5 (25%)	15 (75%)
Seborrhoeic keratosis	17 (3.51%)	7 (41.18%)	10 (58.82%)
Solar lentigo	10 (2.06%)	0	10 (100%)
Periocular hypermelanosis	10 (2.06%)	3 (30%)	7 (70%)
Frictional melanosis	8 (1.65%)	1 (12.5%)	7 (87.5%)
Riehl’s melanosis	7 (1.44%)	0	7 (100%)
Actinic lichen planus	7 (1.44%)	3 (42.86%)	4 (57.14%)
Dermatosis papulosa nigra	6 (1.24%)	1 (16.67%)	5 (83.33%)
Naevus of Ota	4 (0.82%)	0	4 (100%)
Becker’s naevus	4 (0.82%)	4 (100%)	0
Pigmented basal cell carcinoma	2 (0.41%)	1 (50%)	1 (50%0
Peribuccal pigmentation of Brocq	2 (0.41%)	0	2 (100%)
Verrucous epidermal naevus	1	0	1 (100%)
Hori’s naevus	1	0	1 (100%)
Exogenous ochronosis	1	0	1 (100%0
**Total**	**485**	**104 (21.44%)**	**381 (78.56%)**

**Fig. 1:**
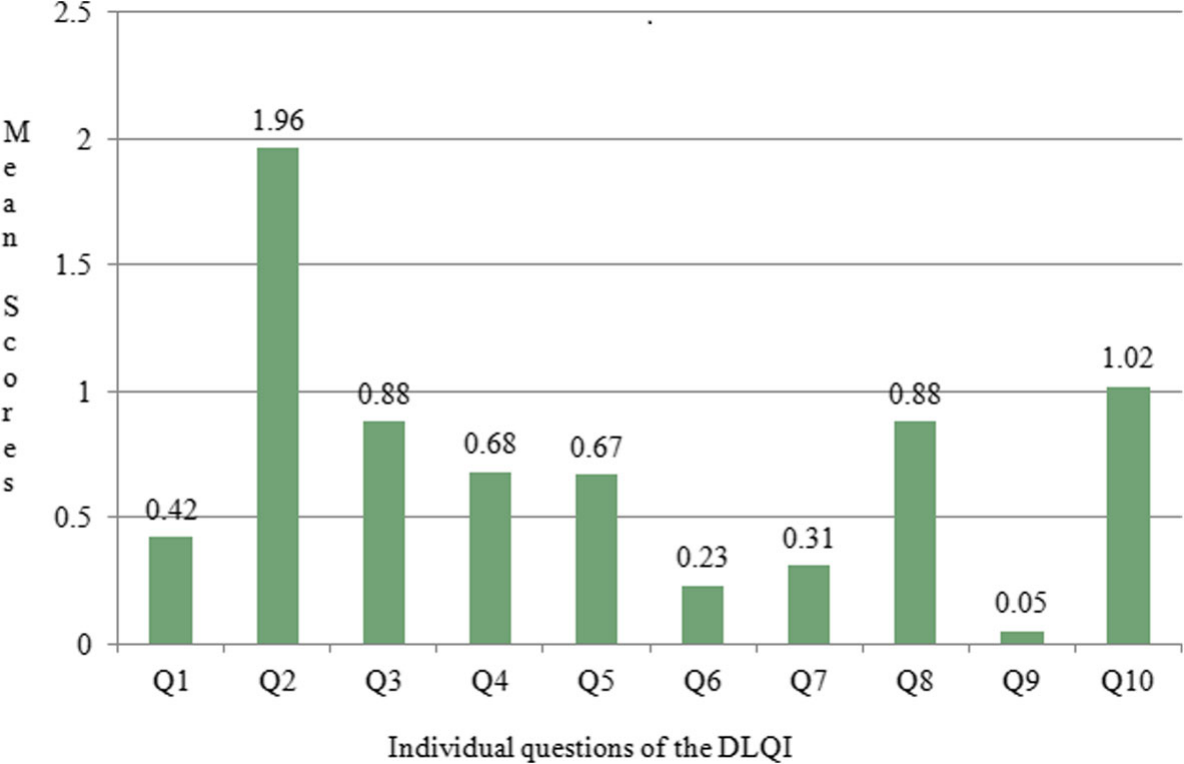
Graph depicting the mean scores in individual questions of the DLQI

The highest DLQI score was observed in patients having melasma with steroid induced rosacea-like dermatitis (13.47 ± 1.33) and the lowest in patients with ephelides (2.45 ± 1.40) (Table 2). An analysis based on individual questions revealed that the second question recorded the highest mean score (1.96 ± 0.78) while the ninth recorded the lowest (0.05 ± 0.22) (Fig. 1).

**Table 2:** Comparison of mean DLQI scores in different facial melanoses

Comparison group	Diagnosis	DLQI (Mean ± SD)	***p*** value
Melasma and melasma with steroid induced rosacea-like dermatitis	Melasma	6.81 ± 1.40	*p* < 0.001
	Melasma with steroid induced rosacea-like dermatitis	13.54 ± 1.30	
Naevi	Compound naevus	3.12 ± 1.30	*p* = 0.266
	Naevus of Ota	3.75 ± 0.96	
	Hori’s naevus	4	
	Becker’s naevus	4 ± 0.82	
	Verrucous epidermal naevus	7	
Secondarily induced melanoses	Acne induced hyperpigmentation	7.73 ± 1.64	*p* = 0.113
	Frictional melanosis	7.94 ± 1.56	
	Postinflammatory hyperpigmentation	8.25 ± 1.49	
	Riehl’s melanosis	8.57 ± 2.94	
	Exogenous ochronosis	10	
Photo induced or photo aggravated dermatoses	Ephelides	2.45 ± 1.40	*p* < 0.001
	Seborrhoeic keratosis	3.41 ± 1.23	
	Solar lentigo	5.10 ± 2.18	
	Actinic lichen planus	7.71 ± 1.98	
	Ashy dermatosis	8.69 ± 2.24	
	DLE	9.04 ± 1.80	
Other facial melanoses	Periocular hypermelanosis	4 ± 1.41	
	Pigmented basal cell carcinoma	4 ± 1.41	
	Dermatosis papulosa nigra	4.33 ± 1.51	
	Peribuccal pigmentation of Brocq	7.50 ± 0.71

**Fig. 2:**
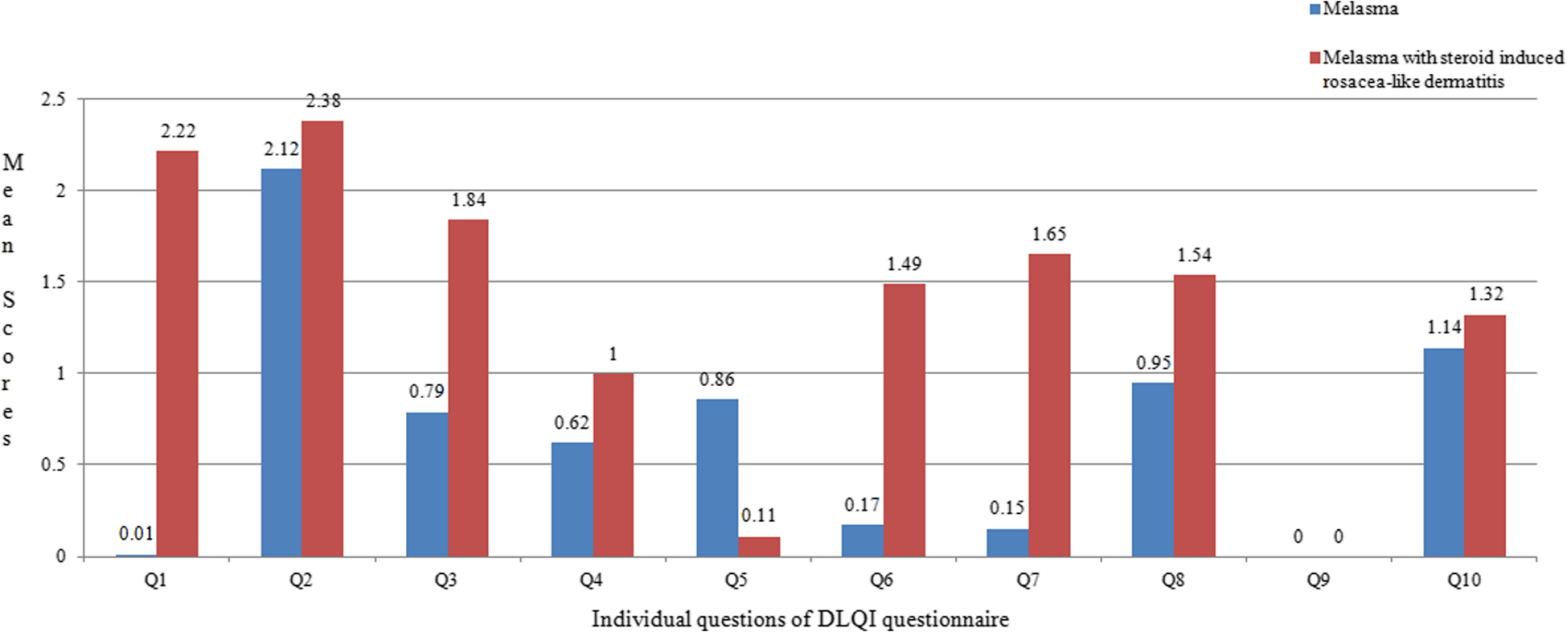
Graph depicting the mean scores in individual questions of the DLQI in participants with melasma and melasma with steroid induced rosacea-like dermatitis

There was a significant difference in DLQI between melasma (6.78 ± 1.34) and melasma with steroid induced rosacea-like dermatitis (13.47 ± 1.33) (*p* < 0.001) (Table 2). Mean DLQI scores for each question revealed that highest score was given for the second question (personal feelings) and lowest for the ninth question (sexual difficulties) (Fig. 2).

Three comparison groups were further analyzed. The first group included various types of naevi, the second group included those conditions that fell under the heading of secondarily induced melanoses (acne induced pigmentation, post-inflammatory hyperpigmentation, Riehl’s melanosis, frictional melanosis and exogenous ochronosis). The third group on the other hand consisted of photo induced or aggravated dermatoses (ashy dermatosis, DLE, ephelides, seborrhoeic keratosis and solar lentigo).

All the conditions that fall under naevi category were associated with a small effect on quality of life except for verrucous epidermal naevus (DLQI = 7) (*p* = 0.266) (Table 2). All the secondarily induced melanoses had a moderate effect on quality of life (Table 2). The highest score was recorded in exogenous ochronosis (10) and the lowest in acne induced pigmentation (7.73 ± 1.64) (*p* = 0.113). In the photo-induced or photo-aggravated facial melanoses group, the highest mean DLQI score was observed in DLE (9.04 ± 1.80) and the lowest in patients with ephelides (2.45 ± 1.40) (p = < 0.001).

## Discussion

Facial melanoses are among the most common diagnoses seen in dermatology outpatient clinic, evidenced by the 672 cases seen in our outpatient department in one year. Previous studies have reported the prevalence of pigmentary disorders as a total burden of skin diseases. Here, we describe the underlying aetiologies of these disorders and provide an assessment of their relative impact on quality of life.

These disorders having abnormal pigmentation in the most visible part of the body are associated with psychosocial and cosmetic challenges. In this study, the highest DLQI was observed in melasma patients having steroid induced rosacea-like dermatitis, suggesting that this condition is associated with a very large impact on quality of life. The magnitude of impact, combined with the relatively high frequency of this diagnosis (a total of 150 patients with steroid induced rosacea-like dermatitis visited our outpatient department during the duration of the study), highlights the impact of this avoidable and preventable condition. In addition to cosmetic impacts, conditions such as steroid induced rosacea-like dermatitis, DLE, frictional melanosis and Riehl’s melanosis are often accompanied by symptoms of pruritus, burning or flushing which further impacts on life quality.

Potent topical corticosteroids in combination with topical antifungals, antibiotics, bleaching agents or topical retinoids are easily available in pharmacies all over Nepal. The salespersons in these pharmacies dispense these combination creams as “Ram Vaan” or “the divine weapon” for the treatment of all dermatological conditions including melasma. However, effective therapies for melasma, such as topical adapalene, tretinoin, azelaic acid, low dose hydroquinone, chemical peels, topical and oral tranexamic acid [3, 13, 14]. are not commonly available to patients outside of major cities in Nepal.

Prior studies have documented the frequent use of steroids for the management of pigment disorders among patients in India and Nepal [15, 16]. This previous study in Nepal also investigated the clinical patterns of steroid induced rosacea-like dermatitis and revealed that steroid medications were purchased and applied without prescription in approximately three quarters (74.4%) of all patients. Most of the participants used the drugs based on the recommendation of friends (38.5%) or pharmacists (20.5%) [16].

Two asymptomatic conditions, namely ashy dermatosis and peribuccal pigmentation of Brocq also had a moderate impact on the quality of life of the participants in this study. Ashy dermatosis is characterized by development of gradually increasing grayish macules on the face and trunk. The evolution is that of gradual progression without spontaneous improvement [17]. Available treatment options are limited and often not effective, which may have contributed to the higher impairment on quality of life [17]. Peribuccal pigmentation of Brocq is a specific type of facial pigmentation involving the peri-oral region with sparing of the vermillion border. The condition may fade gradually but can be persistent and the patients need to be assured about the benign nature of the condition.

Frictional melanosis, Riehl’s melanosis and post-inflammatory hyperpigmentation all had a moderate impact on the quality of life. Patients should be counselled about the nature of, and factors that exacerbate the condition (friction in the case of frictional melanosis and cosmetics in Riehl’s melanosis). Post-inflammatory hyperpigmentation can follow mild trauma or burns especially in females who work in the kitchen. They can be a source of embarrassment for the sufferers and at the same time, the colour intensity can increase due to friction from clothing [18].

This study had some important strength including the systematic inclusion of all patients presenting to a large dermatology clinic in Nepal. However, there were also some notable limitations to the study. Given that this study was cross sectional, we did not capture longitudinal data. This may have been particularly important for conditions such as actinic lichen planus, ephelides and DLEs, all of which are known to have waxing and waning courses. Similarly, the population seeking care in our hospital based study may not be representative of the general population. Many people with facial pigmentary conditions may choose not to visit a clinic for their condition. Another limitation of our study is the failure to assess the association between DLQI and Fitzpatrick skin type. The common Nepali skin type (Fitzpatrick’s type III and IV) tans easily resulting in increased prevalence of photo induced and photo aggravated dermatoses [6]. A comparison of DLQI based on Fitzpatrick skin type could have provided further insight on the impact of these facial melanoses on quality of life.

Although the DLQI questionnaire is a well validated tool to assess disease induced impairment in quality of life, a few questions in this questionnaire may not have been suitable for our population. Nepalese farmers residing in rural areas usually do not have hobbies, leisure activities nor are active participants in sports. Similarly, questions assessing the impact of the disease on sexual activity recorded the lowest score, which may be a function of reporting bias due to reluctance on the part of the participants to give an honest answer to this question. An improved and validated questionnaire could be useful in addressing aspects relating to social discrimination, suspicion of leprosy or cancer, association with blood impurities and the financial burden of treatment.

## Conclusion

Facial melanoses are frequent and important conditions that account for a large number of patient care seeking visits and are associated with substantial impacts on quality of life. Some preventable conditions, such as melasma with steroid induced rosacea-like dermatitis and frictional melanosis, have high disease induced impairment on quality of life. These data suggest that the appropriate diagnosis, management and prevention of these conditions may have a substantial impact on the health and well-being of the population.

## Data Availability

The raw data supporting our findings has been made publicly viewable and can be accessed from the following site: https://www.kaggle.com/bibushamatya/facial-melanoses-dataset-bibush-amatya.

## Abbreviations

NMCTH: Nepal Medical College and Teaching Hospital
DLQI: Dermatology Life Quality Index
SPSS: Statistical Package for Social Sciences
DLE: Discoid lupus erythematosus
